# Evolution of developmental signalling in Dictyostelid social amoebas

**DOI:** 10.1016/j.gde.2016.05.014

**Published:** 2016-08

**Authors:** Pauline Schaap

**Affiliations:** School of Life Sciences, University of Dundee, DD15EH Dundee, UK

## Abstract

Dictyostelia represent a tractable system to resolve the evolution of cell-type specialization, with some taxa differentiating into spores only, and other taxa with additionally one or up to four somatic cell types. One of the latter forms, *Dictyostelium discoideum*, is a popular model system for cell biology and developmental biology with key signalling pathways controlling cell-specialization being resolved recently. For the most dominant pathways, evolutionary origins were retraced to a stress response in the unicellular ancestor, while modifications in the ancestral pathway were associated with acquisition of multicellular complexity. This review summarizes our current understanding of developmental signalling in *D. discoideum* and its evolution.

**Current Opinion in Genetics & Development** 2016, **39**:29–34

This review comes from a themed issue on **Developmental mechanisms, patterning and evolution**

Edited by **Detlev Arendt** and **Cassandra Extavour**

For a complete overview see the Issue and the Editorial

Available online 16th June 2016

**http://dx.doi.org/10.1016/j.gde.2016.05.014**

0959-437X/© 2016 Published by Elsevier Ltd. This is an open access article under the CC BY license (http://creativecommons.org/licenses/by/4.0/).

## Introduction

The Dictyostelid social amoebas evolved multicellularity 0.6 billion years ago as a strategy to survive starvation as aerially borne spores, supported by dead stalk cells [1]. Their last common ancestors are unicellular amoebozoa that encyst individually under starvation stress, or alternatively form a single spore that produces its own acellular stalk. In the course of evolution, cell types diversified further into basal disc cells that anchor the stalk to the substratum and upper- and lower cup cells that secure the spore mass to the stalk. This form of sorocarpic (fruiting body) multicellularity is not unique, but rather evolved at least eight times independently in several eukaryote kingdoms and in prokaryotes [2]. However, in Dictyostelia it reached its highest level of organization with efficient cellular aggregation and highly coordinated morphogenesis.

The developmental programme has been most thoroughly studied in *Dictyostelium discoideum*, a robust laboratory model that uses cAMP as a chemoattractant for aggregation. The development of genetic transformation, gene knock-out, targeted mutagenesis and high resolution imaging techniques in the '80 and '90s, make it the organism of choice for research into fundamental problems in cell biology and developmental biology [3]. More recently, it has also gained popularity for studies into social conflict [4], prey–predator interactions [5] and evolution of multicellular complexity [6]. In this review, I will first describe the developmental signalling mechanisms that control the life cycle of *D. discoideum* and next summarize studies aimed to elucidate in which order specific aspects of complexity evolved, and how this was associated with innovations in intercellular communication.

## *D. discoideum* developmental control is dominated by cAMP

**Figure 1:**
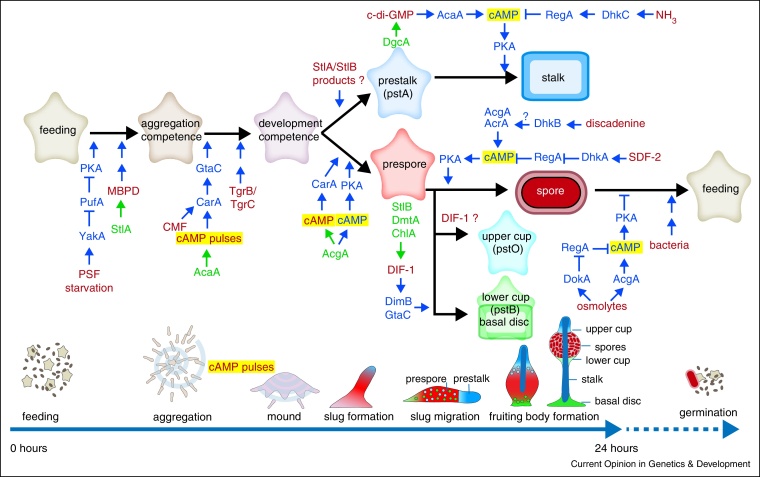
Developmental signalling during the *D. discoideum* life cycle. During their 24 h life cycle, starving amoebas aggregate by secreting and relaying cAMP pulses to form multicellular mounds. The mound tip continues to emit cAMP pulses attracting cells from underneath, which push the tip upwards to form a slug. In the slug cells differentiate into precursors of spore and stalk, basal disc and upper and lower cup cells. Upon initiation of fruiting body formation, tip cells differentiate into stalk cells and move downwards. Most remaining cells move up the stalk and differentiate into spores and upper and lower cup cells. Cells that remain on the substratum differentiate into a basal disc. Stalk and basal disc cells share a similar highly vacuolated phenotype with a cellulose wall, while spores have condensed cytosol and a three-layered cellulosic wall that is also protein-rich. The environmental and secreted signals that control these life cycle transitions and the differentiation of amoebas are shown in red, with processes regulated by cAMP highlighted in yellow. The enzymes that synthesize secreted signals are shown in green, while proteins and small molecules that mediate intracellular signal processing are shown in blue. Blue arrows and t-crosses denote stimulatory and inhibitory effects, respectively, while double blue arrows signify that the mode of action of the signal is unknown. *Abbreviations*: AcaA: adenylate cyclase A; AcgA: adenylate cyclase G; AcrA: adenylate cyclase R; cAMP: 3′-5′-cyclic adenosine monophosphate; CarA: cAMP receptor 1; c-di-GMP: 3′,5′-cyclic diguanylic acid; ChlA: flavin-dependent halogenase Chlorination A; CMF: conditioned medium factor; DgcA: diguanylate cyclase A; DhkA: histidine phosphatase A; DhkB: histidine kinase B; DhkC: histidine kinase C; DIF-1: differentiation inducing factor 1; DimB: transcription factor DIF-insensitive mutant A; DmtA: des-methyl-DIF-1 methyltransferase; DokA: osmosensing histidine phosphatase; GtaC: GATA-binding transcription factor C; MBPD: 4-methyl-5-pentylbenzene-1,3-diol; NH_3_: ammonia; PKA: cAMP-dependent protein kinase; PSF: prestarvation factor; PufA: pumilio RNA binding protein; RegA: cAMP phosphodiesterase with response regulator; SDF-2: spore differentiation factor 2; StlA: polyketide synthase Steely A; StlB: polyketide synthase Steely B; Tgr: transmembrane, IPT, IG, E-set, repeat protein; YakA: DYRK family protein kinase.

The identification of cAMP as the *D. discoideum* chemoattractant was soon followed by reports that cAMP also regulates cell differentiation. More regulatory signals were identified in later years (see [7^•^] for a comprehensive review) and current knowledge of the signals and pathways that regulate the developmental programme is summarized in Figure 1. The transition from growth to multicellular development occurs when the bacterial food runs out and amoeba density is high. Amoebas assess their own density by secreting a glycoprotein, PSF (prestarvation factor) at a constant rate [8]. The combination of starvation and high PSF induces expression of the protein kinase YakA [9]. YakA inhibits binding of the translational repressor PufA to the 3′ end of the catalytic subunit of cAMP-dependent protein kinase (*PkaC*) [10]. PkaC is now translated and triggers basal expression of genes that are required for aggregation, such as the cAMP receptor *carA*, the adenylate cyclase *acaA* and the extracellular cAMP phosphodiesterase *pdsA* [11]. In addition to PSF, amoebas secrete a protein, CMF, (conditioned medium factor), which is needed for CarA-mediated signal transduction [12], and synthesize the polyketide MPBD (4-methyl-5-pentylbenzene-1,3-diol), that enhances expression of aggregation genes [13].

CarA, AcaA and PdsA are part of the network that generates the secreted pulses of cAMP, which cause cells to aggregate into mounds. The mound tip continues to emit cAMP pulses, which, by attracting cells from underneath, cause the cell mass to project upwards and form the slug [14]. The cAMP pulses also induce intermittent translocation of the transcription factor GtaC to the nucleus [15^••^], which induces expression of genes that are required during and after aggregation. Among these genes are *carA*, *acaA*, *pkaR* and *regA* and the cell adhesion genes *csaA*, *tgrB1* and *tgrC1*. TgrB1 and TgrC1 are heterophilic cell adhesion proteins that upon interaction induce competence for post-aggregative cell differentiation. Because the Tgr proteins are highly variable between species, they also act to avoid genetic conflict by preventing non-related strains from participating in the same fruiting body. Such cells can cheat the host by differentiating mainly into spores and not stalk cells [16^••^].

After aggregation, a second adenylate cyclase, AcgA, is translationally upregulated in the posterior of the slug, where increased secreted and intracellular cAMP induces prespore differentiation [17]. The prespore cells express the enzymes StlB, DmtA and ChlA, which synthesize the chlorinated cyclohexanone DIF-1 [18, 19, 20]. DIF-1 induces differentiation of some posterior cells into pstO cells, which later form the upper cup of the spore mass, and others into pstB cells, which will form the lower cup and basal disc [21]. A polyketide produced by either StlB or StlA, which is neither DIF-1 nor MBPD, is required for expression of pstA genes at the anterior of the prestalk region. However, neither StlA nor StlB are required for stalk formation [19, 22].

The signal for stalk cell differentiation is c-di-GMP, which is synthesized by diguanylate cyclase A in prestalk cells [23]. Diguanylate cyclases were previously only found in prokaryotes, where c-di-GMP mediates the effect of a range of stimuli that induce biofilm formation and other cellular responses [24]. c-di-GMP strongly activates AcaA, which in slugs is predominantly expressed at the utmost tip to coordinate morphogenetic cell movement. Increased intracellular cAMP then acts on PKA to activate stalk gene expression [25].

PKA activation also induces spore maturation and prevents spores from germinating in the absence of food [26, 27]. Both AcgA and a third adenylate cyclase, AcrA, [28] synthesize cAMP in spores, but cAMP hydrolysis by the phosphodiesterase RegA crucially regulates the appropriate timing of spore maturation. RegA is activated by phosphorylation of its N-terminal response regulator by sensor histidine kinases/phosphatases (SHK/Ps) [29]. Most signals that control spore and stalk differentiation act on SHK/Ps to either activate or inhibit RegA. Prestalk cells cleave the protein AcbA, released by prespore cells, to yield the peptide SDF-2 [30], which acts on the SHP DhkA to dephosphorylate RegA and so raise cAMP levels and activate PKA. The high ambient osmolarity of the spore head acts on the osmosensor of AcgA to activate cAMP synthesis and on the SHP DokA to inhibit RegA, thereby activating PKA in a two-pronged attack [27, 31]. Discadenine, a cytokinin released by prestalk cells, activates spore maturation and prevents spore germination by activating the SHK DhkB, which is thought to increase AcrA activity [7^•^].

Stalk cell differentiation is inhibited in the slug stage by ammonia, the product of protein degradation. Ammonia activates the SHK DhkC, which activates RegA and thereby inhibits PKA [32]. Ammonia is lost by diffusion from the aerially projecting fruiting body tip of the early fruiting body, thus lifting PKA inhibition. The redundancy in the pathways regulating spore and stalk formation ensures that the encapsulated spores and stalk cells form at the right time and place, without impacting on fruiting body morphogenesis, which requires amoebae to remain motile.

## Developmental cAMP signalling is derived from a unicellular stress response

**Figure 2:**
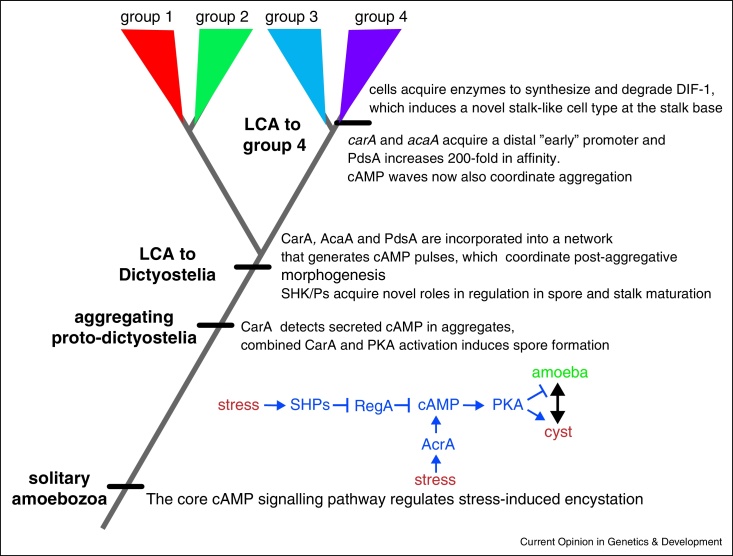
Evolution of developmental signalling from a stress response. Dictyostelia are members of the mostly unicellular kingdom of Amoebozoa. They can be subdivided into two branches each containing two major groups [6]. Comparative analysis of cAMP and DIF-1 signalling across the phylogeny suggests a scenario for the evolution of developmental signalling. cAMP was first used as intermediate for stress-induced encystation in unicellular amoebozoa, with stress acting on sensor histidine phosphatases to inhibit RegA, causing cAMP produced by AcrA, to increase and activate PKA and thereby encystation [34•, 35]. During Dictyostelid evolution sensor histidine kinases and phosphatase acquired novel roles in sensing developmental signals that control timely spore and stalk maturation. Early aggregating prototypes use secreted cAMP accumulating in aggregates as a signal for spore formation [39]. In early Dictyostelia, an emerging network of CarA, AcaA and PdsA produces cAMP pulses to coordinate fruiting body morphogenesis [38, 43]. Finally, group 4 acquires DIF-1 as a signal for basal disc formation, while addition of distal ‘early’ promoters to *carA* and *acaA* genes, and increased affinity of PdsA enables the use of cAMP as chemoattractant for aggregation in group 4 [38, 43, 48].

In recent years, comparative studies have been undertaken to retrace the evolutionary history of cAMP signalling. Dictyostelia can be subdivided into two major monophyletic branches, each containing two major sister groups (Figure 2) [6]. *D. discoideum* resides in group 4 among other species that use cAMP as chemoattractant, have a well-proportioned prestalk/prespore pattern, and build robust solitary fruiting bodies, supported by basal discs. This is distinct from groups 1–3, which use other chemoattractants for aggregation, form small clustered fruiting bodies without supporting discs, and form the stalk by redifferentiation of prespore cells. Many group 1–3 species, such as *Polysphondylium pallidum*, additionally retain the ancestral survival strategy of encystment as individuals [6, 33•]. The enzymes AcrA, AcgA, RegA and PKA are conserved throughout Dictyostelia, and except for AcgA, also in unicellular Amoebozoa [34•, 35]. Knock-out of *P. pallidum PKA* or double knock-out of *AcrA* and *AcgA* prevents encystation, while knock-out of *RegA* causes precocious encystation, while amoebas are still feeding. PKA is also required for *P. pallidum* fruiting body formation, but AcrA and AcgA are not needed for spore or stalk formation, with their roles likely taken over by three copies of the *AcaA* gene. RegA inhibition also causes precocious encystation in the unicellular amoebozoan *Acanthamoeba castellanii* [34•, 35]. Combined, these data indicate that the roles of intracellular cAMP, PKA and RegA in *D. discoideum* spore and stalk maturation are evolutionarily derived from an ancestral role in amoebozoan encystation. The genomes of free-living amoebozoa contain many SHK/Ps that in *D. discoideum* regulate RegA activity [36, 37]. In the unicellular amoebas these SHK/Ps may sense environmental factors, such as drought stress or food, and act on RegA to increase or decrease cAMP levels, respectively, and thereby cause encystation or excystation.

The enzymes AcaA and PdsA and the cAMP receptor CarA, which are important for extracellular cAMP signalling, are conserved in Dictyostelia, but, apart from PdsA, not in unicellular Amoebozoa. CarA is only expressed after aggregation in groups 1–3, and deletion of *carA* or *pdsA* in *P. pallidum* disrupts fruiting body morphogenesis, but not aggregation [38, 39]. The role of extracellular cAMP in coordinating aggregation is therefore derived from an ancestral role in controlling post-aggregative morphogenesis. Loss of *carA* in *P. pallidum* also prevents cAMP-induced prespore differentiation, causing cysts to differentiate in the ‘spore’ head [39]. *Dictyostelium* cells secrete most of their cAMP [40]. While starving, extracellular cAMP levels will therefore particularly increase in the confined cellular interstices of the aggregate. For early Dictyostelia, this increase in extracellular cAMP may have acted as the cue to form spores, when aggregated, and not cysts.

The extracellular cAMP phosphodiesterase PdsA and its secreted inhibitor PdiA affect the kinetics of cAMP wave propagation during *D. discoideum* aggregation, favouring spiral over concentric waves. Spiral waves organize larger territories and hence give rise to large aggregates and robust fruiting bodies [41, 42]. PdiA belongs to a matrix protein family, but only has true orthologs in group 4, while groups 1–3 PdsAs have a 200-fold lower affinity for cAMP than *D. discoideum* PdsA, and only partially restore aggregation of a *D. discoideum pdsA null* mutant [43]. The use of cAMP as chemoattractant in group 4 and its association with robust aggregation and fruiting body formation therefore depended both on changes in PdsA protein function, recruitment of a matrix protein as a PdsA inhibitor and changes in *carA* gene expression.

## DIF-1 signalling causes increased cell-type specialization

DIF-1 is a secreted chlorinated polyketide, that induces differentiation of stalk-like cells *in vitro* [44]. However, cells lacking either of its biosynthetic enzymes StlB, DmtA and ChlA still form normal stalks, but no longer form the basal disc [18, 19, 20]. *StlB*, *dmtA* and *chlA* genes are conserved throughout Dictyostelia, but a group 2 *dmtA* does not complement the *D. discoideum dmta-*mutant [45], and a single tested group 3 species cannot synthesize DIF-1 [46]. The DIF-degrading dechlorinase DcrA is also unique to group 4, supporting biochemical evidence that group 3 species cannot dechlorinate DIF-1 [47]. These data indicate that DIF-1 signalling is unique to group 4. Because it induces basal disc cells, also unique to group 4, it is that likely that modification of its synthetic pathway caused increased cell type specialization in this group.

## Conclusions

The comparative studies allow reconstruction of a tentative narrative about the evolutionary origins of developmental signalling in Dictyostelia, and innovations that occurred at successive stages of their evolution (Figure 2). This narrative assigns a hierarchical structure to the manifold roles of cAMP in modern Dictyostelia, with the second messenger role of cAMP in induction of spore formation at the top of the hierarchy. While corroborating evidence for several proposed events is still required, the comparative approach adds a depth of understanding to the underlying logic of current signalling complexity that would be almost impossible to acquire by a single organism approach. The comparative approach can be used for understanding hierarchy in any complex biological network. This is becoming feasible by the rapid increase in phylogeny-wide genome sequences and novel procedures for knock-down, disruption or replacement of genes in a broad range of organisms.

## References and recommended reading

Papers of particular interest, published within the period of review, have been highlighted as:

• of special interest
•• of outstanding interest
